# Association of Serum Biomarkers With Post-Thrombolytic Symptomatic Intracranial Hemorrhage in Stroke: A Comprehensive Protein Microarray Analysis From INTRECIS Study

**DOI:** 10.3389/fneur.2022.751912

**Published:** 2022-01-31

**Authors:** Yu Cui, Yong Zhao, Shao-Yuan Chen, Bao-Ying Sheng, Li-Hua Wang, Wei-Hong Meng, Hui-Sheng Chen

**Affiliations:** ^1^Department of Life Science and Biopharmaceutics, Shenyang Pharmaceutical University, Shenyang, China; ^2^Department of Neurology, General Hospital of Northern Theatre Command, Shenyang, China; ^3^Department of Neurology, Haicheng Hospital of Traditional Chinese Medicine, Haicheng, China; ^4^Department of Neurology, Chinese People's Liberation Army 321 Hospital, Baicheng, China; ^5^Department of Neurology, Jiamusi University First Affiliated Hospital, Jiamusi, China; ^6^Department of Neurology, The Second Affiliated Hospital of Harbin Medical University, Harbin, China

**Keywords:** ischemic stroke, intravenous thrombolysis, symptomatic intracranial hemorrhage, biomarkers, microarray analysis, tPA

## Abstract

**Background:**

Symptomatic intracranial hemorrhage (sICH) after intravenous thrombolysis is closely related to the poor outcome of stroke.

**Aims:**

To determine the serum biomarkers associated with sICH based on the INTRECIS study.

**Methods:**

Enrolled patients with sICH and without any ICH were matched by propensity score matching with the ratio of 1:1. Preset 49 biomarkers were measured by protein microarray analysis. Gene Ontology and Pathway Enrichment Analysis and protein-protein interaction network (PPI) were analyzed in the identified biomarkers.

**Results:**

Of the consecutive 358 patients, eight patients occurred with sICH, which was assigned as an sICH group, while eight matched patients without any ICH were assigned as a Non-sICH group. A total of nine biomarkers were found significantly different between groups, among which the levels of interferon (IFN)-γ and interleukin (IL)-4 were higher, while the levels of C-reactive protein (CRP), glial cell line-derived neurotrophic factor (GDNF), insulin-like growth factor-binding protein (IGFBP)-6, lymphatic vessel endothelial hyaluronan receptor (LYVE)-1, matrix metalloprotein (MMP)-2, plasminogen activator inhibitor (PAI)-1, and platelet-derived growth factor (PDGF)-AA were lower in the sICH group compared with those in the Non-sICH group.

**Conclusions:**

Our finding indicated that baseline serum CRP, GDNF, IFN-γ, IGFBP-6, IL-4, LYVE-1, MMP-2, PAI-1, and PDGF-AA levels were associated with post-thrombolytic sICH in stroke.

## Introduction

Intravenous thrombolysis, with recombinant tissue plasminogen activator (rtPA), is an effective treatment for acute ischemic stroke within 4.5 h of symptom onset (1). Symptomatic intracranial hemorrhage (sICH) is a rare but severe complication following intravenous thrombolysis, which is closely related to disability and death (2). Thus, it is critical to identify the predictive biomarkers for post-thrombolytic sICH.

Previous studies have investigated clinical, radiological, and laboratory risk factors of hemorrhagic transformation in ischemic stroke (3–8). Contributing to proteolysis, oxidative stress, and leukocyte infiltration in hemorrhagic transformation (9), several biomarkers were found associated with hemorrhagic transformation (10), However, serum biomarkers for post-thrombolytic sICH have not been fully determined.

In INtravenous Thrombolysis REgistry for Chinese Ischemic Stroke within 4.5 h of onset (INTRECIS) (11), five centers were pre-designed to consecutively collect blood samples prior to intravenous thrombolysis for additional exploratory research. In the present study, we measured baseline serum levels of 49 well-known biomarkers in patients with thrombolysis, matched with sICH vs. Non-sICH and tried to identify the associated biomarkers with sICH and their interactions through protein microarray analysis.

## Methods

### Study Population and Procedure

From August 2018 to July 2019, patients receiving intravenous thrombolysis within 4.5 h after symptom onset were consecutively screened to collect blood samples prior to thrombolysis from five pre-set stroke centers in the INTRECIS study (registered at ClinicalTrials.gov NCT 02854592). The INTRECIS is a nationwide, multi-center, prospective, and registry study of consecutive adult patients who were eligible for treatment with intravenous thrombolysis within 4.5 h of the onset of symptoms. Details of the study design and results of the primary outcomes have been reported recently (11). The inclusion criteria were that patients received a standard dose of rtPA (0.9 mg/kg, maximum 90 mg; manufacturer: Boehringer Ingelheim) within 4.5 h after the symptoms onset. The exclusion criteria were as follows: (1) patients received urokinase, (2) patients received a non-standard dose of rtPA, (3) patients received an endovascular intervention, (4) patients lacked complete clinical data, and (5) blood samples were not collected prior to intravenous thrombolysis. All the patients and/or their legal guardians gave written informed consent for data collection. According to the presence or absence of sICH, enrolled patients were divided into two groups, namely, (1) sICH group: patients with sICH and (2) Non-sICH group: patients without any ICH. Furthermore, propensity score matching was performed between groups with the ratio 1:1, the caliper of 0.1, and a nearest-neighbor matching strategy, and operated with control factors including age, gender, current smoking, alcohol consumption, systolic blood pressure, diastolic blood pressure, blood glucose, symptom onset-to-treatment time, National Institutes of Health Stroke Scale (NIHSS) score at admission, the Trial of Org 10172 in Acute Stroke Treatment (TOAST) classification (12), previous use of antiplatelet, and medical history.

The baseline characteristics and clinical data of recruited patients were obtained from an electronic database. This included age, gender, current smoking, alcohol consumption, systolic blood pressure, diastolic blood pressure, blood glucose, symptom onset-to-thrombolytic time, NIHSS scores, TOAST classification, previous use of antiplatelet, history of stroke, hypertension, diabetes mellitus, atrial fibrillation, and congestive heart failure. All patients underwent a brain computerized tomography scan at admission and 24 h after intravenous thrombolysis (or earlier when a neurological worsening occurred) to evaluate the presence of intracranial hemorrhage. According to European Cooperative Acute Stroke Study (ECASS)—II definition, sICH was defined as an increase of ≥4 on the NIHSS scores caused by intracranial hemorrhage within 36 h (13).

### Ethics Approval

The study was centrally approved by the Institution Human Research Ethics Committees of General Hospital of Northern Theater Command.

### Blood Sampling and Biomarkers Measurements

About 4 ml of peripheral venous blood samples were collected from each patient just prior to intravenous thrombolysis. The blood samples were centrifuged at 1,000 × g for 10 min at 4°C, and then transferred into a 1.8-milliliter cryotube and stored at −80°C until measurement.

According to the instructions of the manufacturer, pre-customized protein microarray analysis (Raybiotech Inc.) was used to simultaneously detect and quantify the 49 biomarkers in the collected blood samples, which were preset based on published data. Identified biomarkers were defined as those variations with *p* < 0.05, and fold change > 1.20 or <0.83. Functional enrichment analysis and protein-protein interactions (PPI) network were performed to explore the possible mechanism between identified biomarkers.

### Statistical Analysis

Descriptive statistics was performed to compare variables between two groups. Continuous variables with normal distribution were described as means and standard deviation. Continuous variables include age, systolic blood pressure, diastolic blood pressure, blood glucose, symptom onset to thrombolysis time, NIHSS score, and detected serum concentration of biomarkers. The *t*-tests were used to analyze the normally distributed continuous variables. Categorical variables were described as numbers and proportions. Categorical variables included gender, current smoking, alcohol consumption, medical history, previous use of antiplatelet, and TOAST classification. The Pearson χ^2^ tests were used to analyze the categorical variables.

The value of *p* is obtained from the moderated t-statistic with a false discovery rate of adjustment for multiple testing. In all analyses, differences were considered statistically significant with a *p* < 0.05. The free statistical language R (version 3.10.3) was used for the outcomes and graph in the propensity score matching and microarray analysis.

## Results

**Figure 1:**
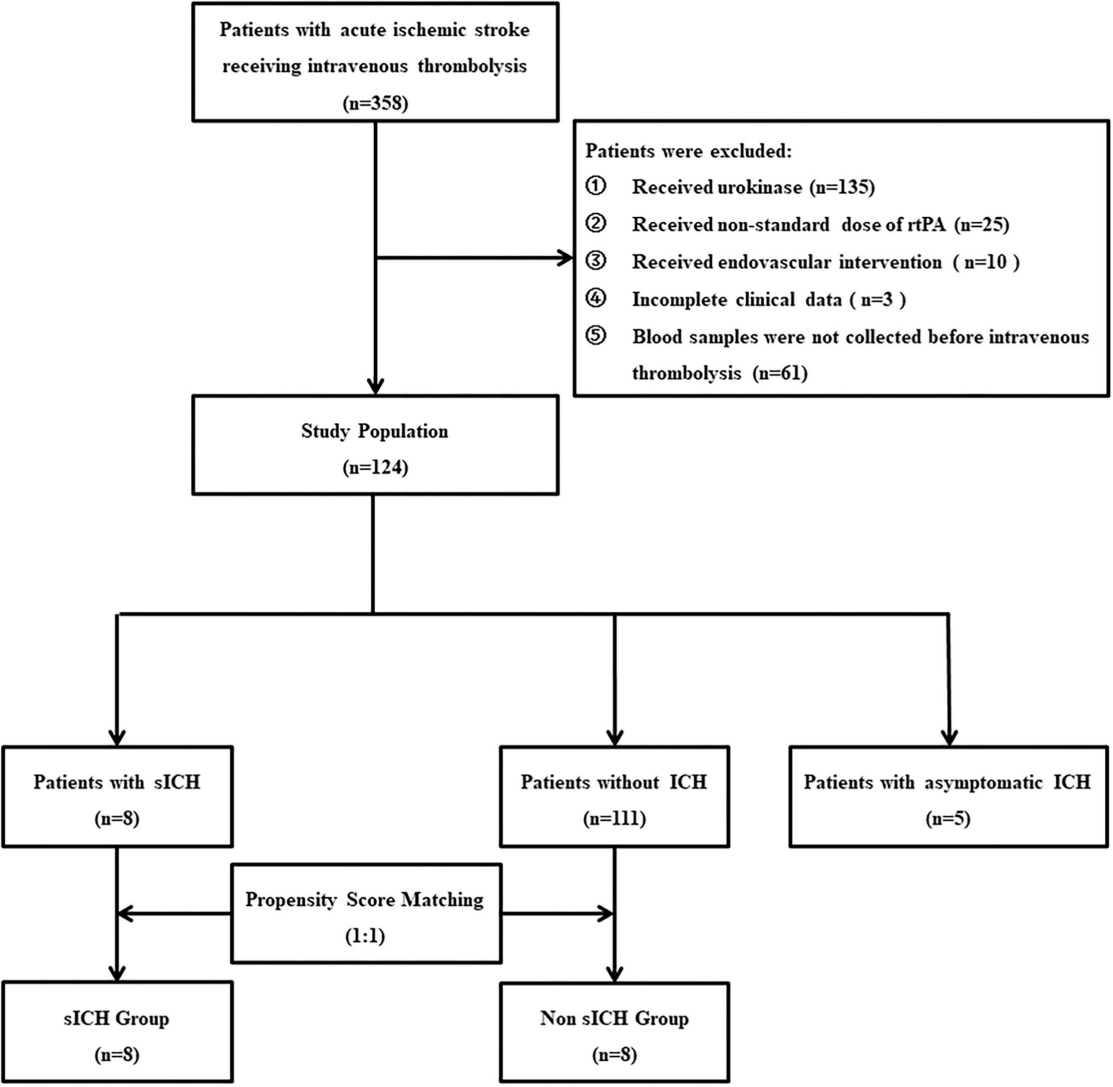
Flow diagram. rtPA, recombinant tissue plasminogen activator; sICH, symptomatic intracranial hemorrhage.

As shown in (Figure 1), 358 patients with thrombolysis were consecutively screened in the present study and 234 patients were excluded for different reasons as follows: 135 patients received urokinase, 25 received a non-standard dose of rtPA, 10 patients received the endovascular intervention, three patients with incomplete clinical data, and 61 patients without blood sample collection. Finally, 124 patients were recruited into the current study, including five patients with asymptomatic intracranial hemorrhage, eight patients with sICH, and 111 patients without any ICH 36 h after intravenous thrombolysis. With the ratio 1:1, eight patients without any ICH and eight patients with sICH were matched to the non-sICH group and sICH group for comparative analysis, respectively (Figure 1). There was no significant difference in the baseline characteristics between the two groups (Table 1).

**Table 1:** Baseline characteristics in symptomatic intracranial hemorrhage (sICH) and Non-sICH groups.

**Variables**	**sICH** **(*n* = 8)**	**Non-sICH** **(*n* = 8)**	***P* Value**
**Demographics**			
Age, years, mean ± SD	64.1 ± 11.4	64.5 ± 9.99	0.945
Gender, male, n (%)	3 (37.5)	3 (37.5)	1.000
Current smoking, n (%)	1 (12.5)	2 (25.0)	0.522
Alcohol consumption, n (%)	1 (12.5)	1 (12.5)	1.000
**Medical history, n (%)**			
Stroke	2 (25.0)	2 (25.0)	1.000
Hypertension	7 (87.5)	6 (75.0)	0.552
Diabetes mellitus	2 (25.0)	2 (25.0)	0.634
Atrial fibrillation	0 (0.0)	1 (12.5)	0.302
Congestive heart failure	3 (37.5)	4 (50.0)	0.614
Previous use of antiplatelet	1 (12.5)	1 (12.5)	1.000
**Baseline scales, mean** **±SD**			
Systolic blood pressure, mmHg	160.3 ± 17.4	150.6 ± 13.7	0.239
Diastolic blood pressure, mmHg	91.3 ± 4.5	88.4 ± 11.9	0.532
Blood glucose, mmol/L	8.99 ± 2.64	7.46 ± 2.10	0.219
Symptom onset to thrombolysis time, min	168.5 ± 64.2	181.3 ± 48.3	0.661
NIHSS score at admission	6.8 ± 5.7	4.8 ± 5.6	0.488
NIHSS score after sICH	27.5 ± 15.0		
TOAST classification, n (%)			0.504
Large artery atherosclerosis	4 (50.0)	3 (37.5)	
Cardioembolism	0 (0.0)	2 (25.0)	
Small artery occlusion	1 (12.5)	1 (12.5)	
Undetermined cause	3 (37.5)	2 (25.0)	

*NIHSS indicates National Institute of Health Stroke Scale; SD, standard deviation; sICH, symptomatic intracranial hemorrhage; TOAST, the Trial of Org 10172 in Acute Stroke Treatment*.

### Biomarkers Identification According to Baseline Serum Levels

**Figure 2:**
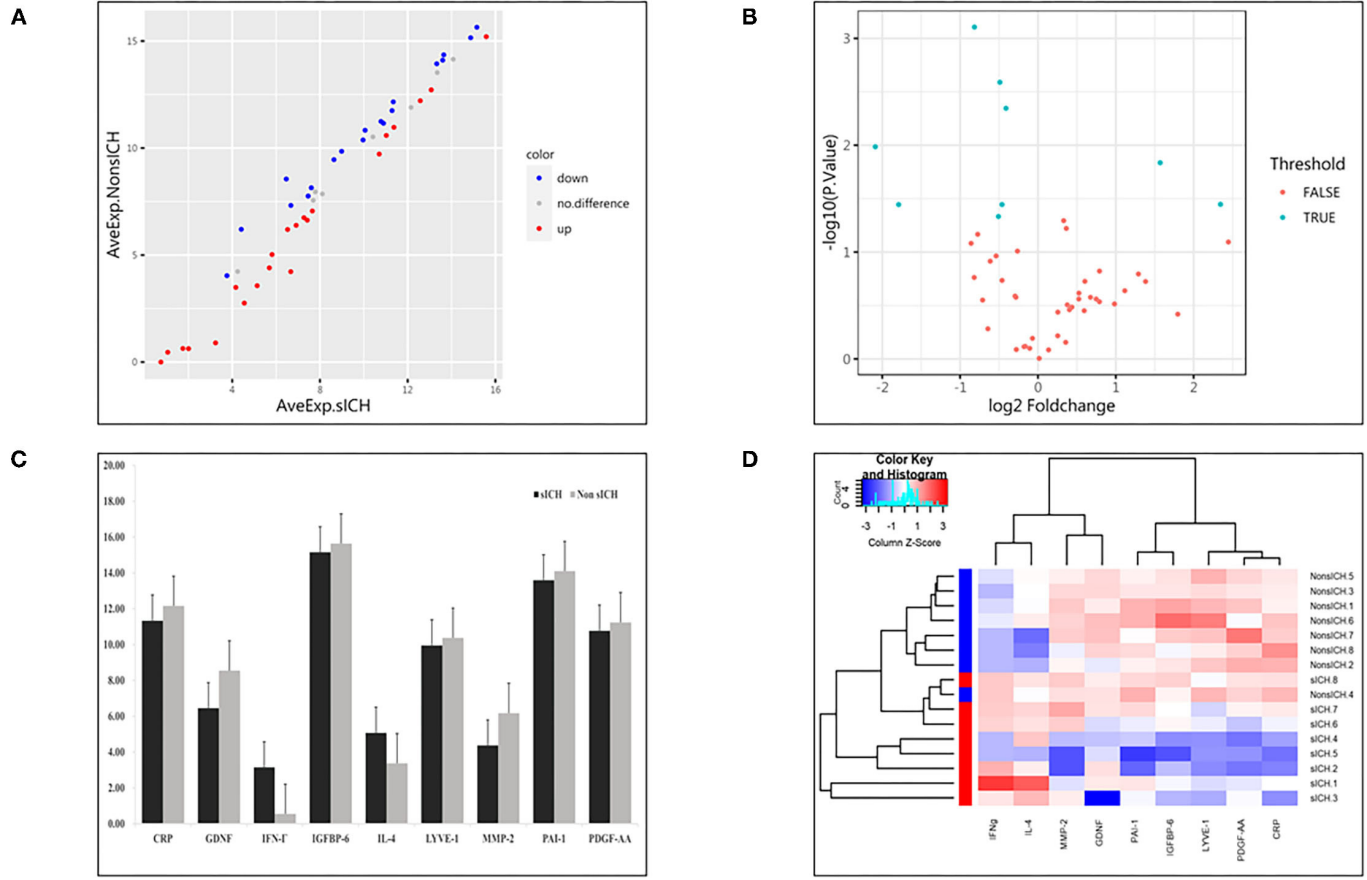
Results of detected biomarkers in the microarray analysis. **(A)** The scatter plot for detected biomarkers; the X-axis represents the average of log_2_ serum levels in the symptomatic intracranial hemorrhage (sICH) group, while the Y-axis represents the average of log_2_ serum levels in Non-sICH group; compared with Non-sICH group, the blue point represents biomarkers with lower serum levels in sICH group, while the red point represents biomarkers with higher serum levels. sICH, symptomatic intracranial hemorrhage. **(B)** The volcano plot for detected biomarkers; the X-axis represents the log_2_ fold-change value, while the Y-axis represents the –log_10_ *P* value; the cyan point represents the biomarkers with significant difference, while the red point represents the biomarkers without significant difference. sICH, symptomatic intracranial hemorrhage. **(C)** The column plot for identified biomarkers; the X-axis represents the identified biomarkers, while the Y-axis represents the average of log_2_ serum levels in two groups; the deep color represents the sICH group, while the light color represents the Non-sICH group. CRP, C-reactive protein; GDNF, glial cell line-derived neurotrophic factor; IFN-γ, interferon gamma; IGFBP-6, insulin-like growth factor binding protein 6; IL-4, interleukin 4; LYVE-1, lymphatic vessel endothelial hyaluronan receptor 1; MMP-2, matrix metalloprotein 2; PAI-1, plasminogen activator inhibitor 1; PDGF-AA, platelet-derived growth factor AA; sICH, symptomatic intracranial hemorrhage. **(D)** The heatmap for identified biomarkers; the red color represents biomarkers with higher serum levels, while the blue color represents the biomarkers with lower serum levels; the darker the color, the more significant the difference of biomarkers. CRP, C-reactive protein; GDNF, glial cell line-derived neurotrophic factor; IFN-γ, interferon gamma; IGFBP-6, insulin-like growth factor binding protein 6; IL-4, interleukin 4; LYVE-1, lymphatic vessel endothelial hyaluronan receptor 1; MMP-2, matrix metalloprotein 2; PAI-1, plasminogen activator inhibitor 1; PDGF-AA, platelet-derived growth factor AA; and sICH, symptomatic intracranial hemorrhage.

Compared with the non-sICH group, nine significantly different biomarkers in the sICH group were observed (*p* < 0.05). Patients with sICH showed higher pretreatment serum levels of interferon (IFN)-γ and interleukin (IL)-4, and lower pretreatment serum levels of C-reactive protein (CRP), glial cell line-derived neurotrophic factor (GDNF), insulin-like growth factor-binding protein (IGFBP)-6, lymphatic vessel endothelial hyaluronan receptor (LYVE)-1, matrix metalloprotein (MMP)-2, plasminogen activator inhibitor (PAI)-1, and platelet-derived growth factor (PDGF)-AA than the matched patients in Non-sICH (Table 2). The scatter and volcano plot showed results of all the measured biomarkers (Figures 2A,B). The heatmap and column plot showed the results of the identified biomarkers (Figures 2C,D).

**Table 2:** Detected pretreatment serum levels of identified biomarkers.

**Biomarkers**	**Pretreatment serum levels**		***P* Value**	**Fold change**
	**sICH** **(*n* = 8)**	**Non-sICH** **(*n* = 8)**		
**Mean** **±SD, pg/ml**				
CRP	2765.70 ± 1023.99	4636.24 ± 832.82	0.038	0.57
GDNF	182.23 ± 139.16	420.63 ± 187.63	0.010	0.24
IFN-γ	37.62 ± 82.70	1.98 ± 4.13	0.036	5.08
IGFBP-6	37543.59 ± 9245.23	51973.31 ± 10099.90	0.003	0.71
IL-4	47.77 ± 49.00	14.25 ± 9.01	0.015	2.97
LYVE-1	1002.81 ± 97.83	1331.03 ± 95.88	0.005	0.75
MMP-2	71.71 ± 87.02	80.37 ± 34.63	0.036	0.29
PAI-1	13131.84 ± 4286.61	17774.75 ± 2477.54	0.046	0.70
PDGF-AA	1789.68 ± 349.52	2439.55 ± 311.09	0.036	0.73

*CRP indicates C-reactive protein; GDNF, glial cell line-derived neurotrophic factor; IFN-γ, interferon gamma; IGFBP-6, insulin-like growth factor binding protein 6; IL-4, interleukin 4; LYVE-1, lymphatic vessel endothelial hyaluronan receptor 1; MMP-2, matrix metalloprotein 2; PAI-1, plasminogen activator inhibitor 1; PDGF-AA, platelet-derived growth factor AA; SD, standard deviation; sICH, symptomatic intracranial hemorrhage*.

### Function Enrichment and PPI Network Analysis

**Figure 3:**
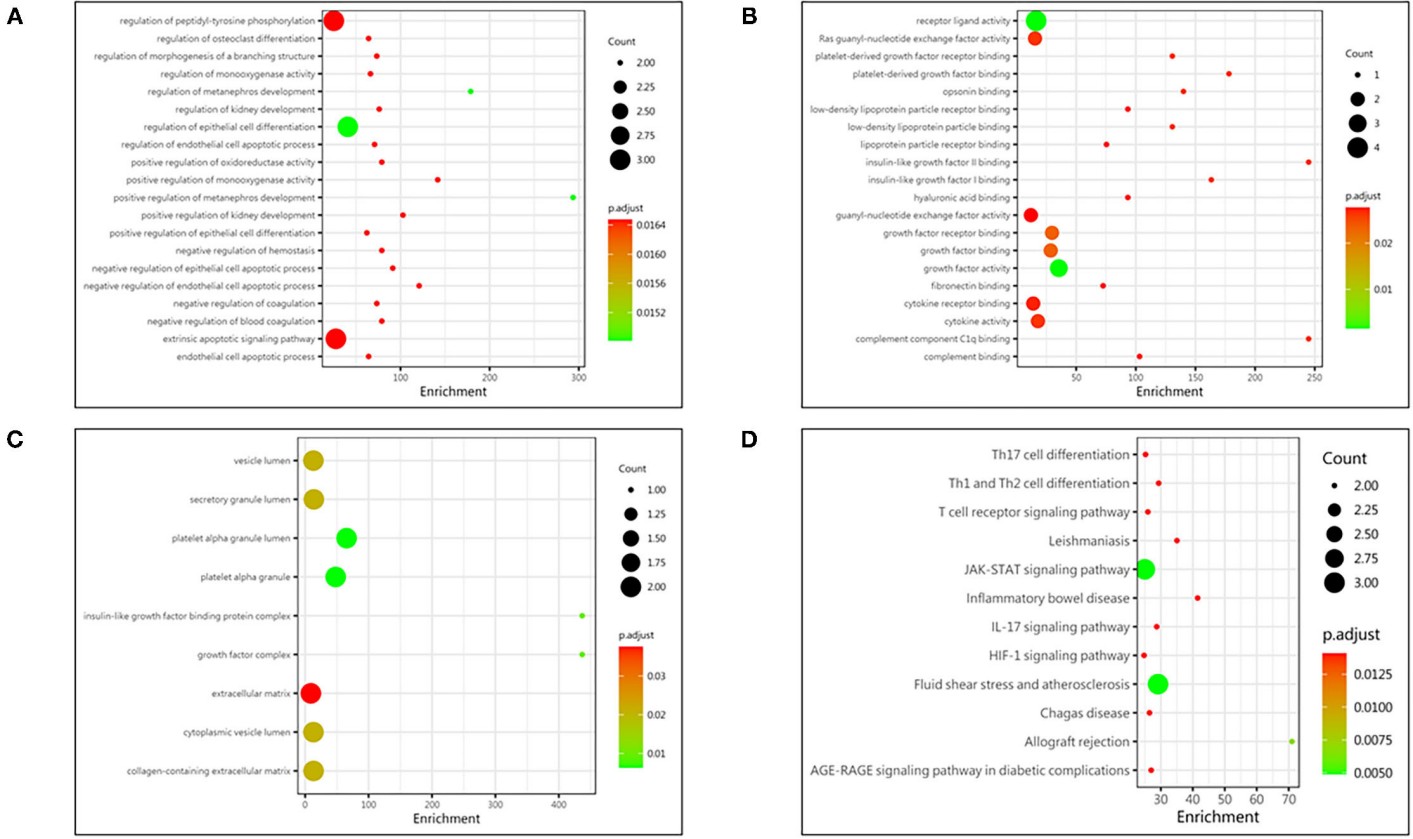
Protein function analysis of identified biomarkers. **(A)** Top 20 significantly enriched biological process of identified biomarkers; the X-axis represents the enrichment, while the Y-axis represents the biological process. **(B)** The molecular function enriched by identified biomarkers; the X-axis represents the enrichment, while the Y-axis represents the molecular function. **(C)** The cellular component enriched by identified biomarkers; the X-axis represents the enrichment, while the Y-axis represents the cellular component. **(D)** The pathway enriched by identified biomarkers; the X-axis represents the enrichment, while the Y-axis represents the pathway. The deeper the color, the larger the *P* value; the larger the circle, the bigger the counts.

The comprehensive Gene Ontology (GO) enrichment analysis was used to gain a deeper insight into the main functions of the identified biomarkers. The GO analysis consisted of biological process, molecular function, and cellular component analysis. The biological process analysis showed that GDNF, IFN-γ, and PAI-1 were mostly included in the regulation of epithelial cell differentiation (Figure 3A). The molecular function analysis showed that GDNF, IL-4, and PDGF-AA were mostly included in the growth factor activity (Figure 3B). The cellular component analysis showed that PAI-1 and PDGF-AA were mostly included in the platelet alpha granule lumen (Figure 3C). The Kyoto Encyclopedia of Genes and Genomes (KEGG) enrichment analysis showed that IFN-γ, IL-4, and PDGF-AA were included in the Jatyrosine Kinase/Signal Transducer and Activator of Transcription (JAK/STAT) signaling pathway (Figure 3D).

**Figure 4:**
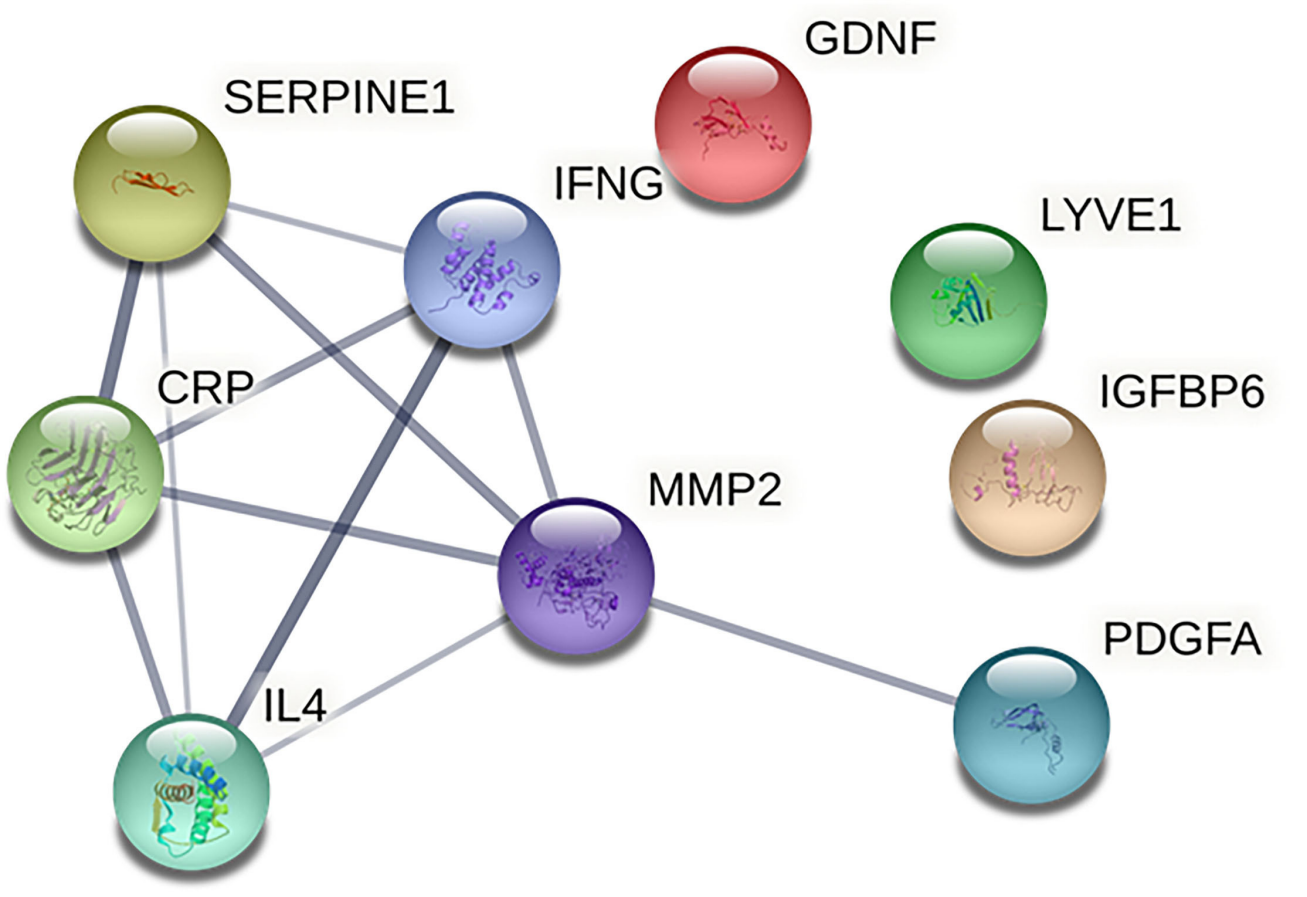
Protein-protein interaction network of identified biomarkers. The CRP indicates C-reactive protein; GDNF, glial cell line-derived neurotrophic factor; IFNG, interferon gamma; IGFBP6, insulin-like growth factor binding protein 6; IL4, interleukin 4; LYVE1, lymphatic vessel endothelial hyaluronan receptor 1; MMP2, matrix metalloprotein 2; SERPINE1, plasminogen activator inhibitor 1; PDGFA, and platelet-derived growth factor AA.

Based on the information of the Search Tool for the Retrieval of Interacting Genes (STRING) database, the PPI network constructed by the above nine identified biomarkers was obtained (Figure 4). The results showed that MMP-2 (degree = 5) could interact with most biomarkers, followed by IFN-γ (degree = 4), PAI-1 (degree = 4), CRP (degree = 4), IL-4 (degree = 4), and PDGF-AA (degree = 1).

## Discussion

In the present study, we found that baseline serum levels of 9 biomarkers were differently expressed in thrombolytic patients with sICH compared with Non-sICH group: higher IFN-γ and IL-4, and lower CRP, GDNF, IGFBP-6, LYVE-1, MMP-2, PAI-1, and PDGF-AA. Of those biomarkers, IGFBP-6, LYVE-1, and PDGF-AA were firstly found associated with sICH.

Up to date, several mechanisms and potential biomarkers for sICH were previously investigated (9, 10). In the present study, we found that IFN-γ and IL-4 were higher in the patients with sICH, while MMP-2, PAI-1, CRP, and GDNF were lower in the patients with sICH, compared with Non-sICH patients. Of those biomarkers, higher IFN-γ and lower PAI-1, CRP, and GDNF seem plausible based on their possible effects as previously reported. For example, the role of lower PAI-1 and CRP in the unbalance between fibrinolysis and thrombosis (14, 15) is the contribution of higher IFN-γ to blood-brain barrier disruption (16) and the neuroprotective effect of GDNF (17). However, higher IL-4 and lower MMP-2 seem unreasonable, given the neuroprotective effect of IL-4 and the blood-brain barrier disruptive effect of MMP-2 (18, 19). We argue that the roles of these biomarkers are very complex, which is further supported by the result of PPI network analysis that there were interactions in IFN-γ, MMP-2, PAI-1, and CRP. Taken together, we inferred that these biomarkers were associated with sICH possibly by blood-brain barrier disruption, unbalance between thrombosis and fibrinolysis, and disruption of endogenous neuroprotection.

For the first time, we found that lower PDGF-AA, IGFBP-6, and LYVE-1 were associated with sICH. The previous studies suggested that lower PDGF-AA may be associated with lower endothelial remodeling degree and higher vascular permeability (20). The IGFBP-6 was found to have an inhibitory role in regard to the actions of IGF-II (21), which was associated with brain injury (22). Collectively, we infer that lower PDGF-AA and IGFBP-6 may indirectly contribute to the development of sICH. As to the association of LYVE-1 with sICH, no related reports were available to date. Thus, the role of these biomarkers in sICH warrants further investigation.

Additionally, we did not detect a significant difference in other preset biomarkers, such as MMP-9, which has been reported to be associated with post-thrombolytic hemorrhagic transformation (23, 24). A possible explanation is that MMP-9 was measured after hemorrhagic transformation in these previous studies while it was measured before hemorrhagic transformation in the current study, which may suggest the dynamic role of MMP-9 in the development of hemorrhagic transformation.

Based on functional enrichment analysis, epithelial cell differentiation, growth factor activity, platelet alpha granule lumen, and JAK/STAT signaling pathway were significantly enriched items. As the initial phase of blood-brain barrier disruption, brain microvascular endothelial cell injury, induced by stroke, played a key role in hemorrhagic transformation (25). Growth factors, like GDNF secreted by pericytes, protected the endothelial cells through enhancing the expression of tight junction protein (26). In addition, blocking JAK/STAT signaling pathway was found to relieve blood-brain barrier disruption (27). Therefore, we inferred that epithelial cell differentiation, growth factor activity, and JAK/STAT signaling pathway may be associated with hemorrhagic transformation through blood-brain barrier disruption.

The strength of this study was to explore the key biomarkers associated with post-thrombolytic sICH through comprehensively screening the preset biomarkers in a prospective cohort and find several new biomarkers, which have never been reported previously. However, we acknowledge that our study has several limitations. First, it is a small-sample study with only eight stroke patients with post-thrombolytic sICH and blood samples. Thus, the results should be further confirmed by a large cohort study with different subtype hemorrhagic transformations, such as asymptomatic and symptomatic intracranial hemorrhage. Second, these identified biomarkers were not confirmed by other methods, such as western blot analysis, enzyme-linked immunosorbent assay, or other *in vivo* experiments. Finally, although the effect of identified biomarkers was independent of other preset biomarkers, the different levels as a result of an acute-phase reaction or previous systemic diseases cannot be ruled out.

## Conclusion

Our finding indicated that baseline serum levels of CRP, GDNF, IFN-γ, IGFBP-6, IL-4, LYVE-1, MMP-2, PAI-1, and PDGF-AA were associated with post-thrombolytic sICH in acute ischemic stroke. The role and predictive value of these identified biomarkers warrant further investigation.

## Data Availability Statement

The datasets presented in this study can be found in online repositories. The names of the repository/repositories and accession number(s) can be found below: https://doi.org/10.5281/zenodo.5801880.

## Ethics Statement

The studies involving human participants were reviewed and approved by Institution Human Research Ethics Committees of General Hospital of Northern Theater Command. The patients/participants provided their written informed consent to participate in this study. Written informed consent was obtained from the individual(s) for the publication of any potentially identifiable images or data included in this article.

## Author Contributions

H-SC designed and reviewed. W-HM supervised the manuscript writing. YC conducted the analyses and drafted the manuscript. YZ, S-YC, B-YS, and L-HW contributed to the implementation of blood sample collection. All authors contributed to the article and approved the submitted version.

## Funding

The study was funded by grants from National Key R&D Program of China (2017YFC1308203), the Project on Research and Application of Effective Intervention Techniques for Chinese Stroke Guidelines from the National Health, and Family Planning Commission in China (GN-2016R0008).

## Conflict of Interest

The authors declare that the research was conducted in the absence of any commercial or financial relationships that could be construed as a potential conflict of interest.

## Publisher's Note

All claims expressed in this article are solely those of the authors and do not necessarily represent those of their affiliated organizations, or those of the publisher, the editors and the reviewers. Any product that may be evaluated in this article, or claim that may be made by its manufacturer, is not guaranteed or endorsed by the publisher.
